# Bioinformatics-Inspired IMU Stride Sequence Modeling for Fatigue Detection Using Spectral–Entropy Features and Hybrid AI in Performance Sports

**DOI:** 10.3390/s26020525

**Published:** 2026-01-13

**Authors:** Attila Biró, Levente Kovács, László Szilágyi

**Affiliations:** 1 Physiological Controls Research Center, Obuda University, 1034 Budapest, Hungary; kovacs@uni-obuda.hu (L.K.); szilagyi.laszlo@uni-obuda.hu (L.S.); 2 Doctoral School of Applied Informatics and Applied Mathematics, Obuda University, 1034 Budapest, Hungary; 3 Department of Physiotherapy, University of Malaga, 29071 Malaga, Spain; 4 Computational Intelligence Research Group, Sapientia Hungarian University of Transylvania, 540485 Targu Mures, Romania; 5 Department of Electrical Engineering and Information Technology, George Emil Palade University of Medicine, Pharmacy, Science and Technology of Targu Mures, Str. Nicolae Iorga, Nr. 1, 540088 Targu Mures, Romania; 6 John von Neumann Faculty of Informatics, Biomatics and Applied Artificial Intelligence Institute, Obuda University, 1034 Budapest, Hungary

**Keywords:** inertial measurement unit, IMU, wearable sensors, running biomechanics, fatigue detection, stride segmentation, spectral analysis, sample entropy, bioinformatics-inspired sequence modeling, mixed-effects modeling, machine learning, hybrid AI, anomaly detection, 1D-CNN

## Abstract

Wearable inertial measurement units (IMUs) provide an accessible means of monitoring fatigue-related changes in running biomechanics, yet most existing methods rely on limited feature sets, lack personalization, or fail to generalize across individuals. This study introduces a bioinformatics-inspired stride sequence modeling framework that integrates spectral–entropy features, sample entropy, frequency-domain descriptors, and mixed-effects statistical modeling to detect fatigue using a single lumbar-mounted IMU. Nineteen recreational runners completed non-fatigued and fatigued 400 m runs, from which we extracted stride-level features and evaluated (1) population-level fatigue classification via global leave-one-participant-out (LOPO) models and (2) individualized fatigue detection through supervised participant-specific models and non-fatigued-only anomaly detection. Mixed-effects models revealed robust and multidimensional fatigue effects across key biomechanical features, with large standardized effect sizes (Cohen’s d up to 1.35) and substantial variance uniquely explained by fatigue (partial R^2^ up to 0.31). Global LOPO machine learning models achieved modest accuracy (55%), highlighting strong inter-individual variability. In contrast, personalized supervised Random Forest classifiers achieved near-perfect performance (mean accuracy 97.7%; mean AUC 0.997), and NF-only One-Class SVMs detected fatigue as a deviation from individual baseline patterns (mean AUC 0.967). Entropy and stride-to-stride variability metrics further demonstrated consistent fatigue-linked increases in movement irregularity and reduced neuromuscular control. These findings show that IMU stride sequences contain highly informative, fatigue-sensitive biomechanical signatures, and that combining bioinformatics-inspired sequence analysis with hybrid statistical and personalized AI models enables both robust population-level insights and highly reliable individualized fatigue monitoring. The proposed framework supports future integration into sports analytics platforms, digital coaching systems, and real-time wearable fatigue detection technologies. This highlights the necessity of personalized fatigue-monitoring strategies in wearable systems.

## 1. Introduction

**Figure 1:**
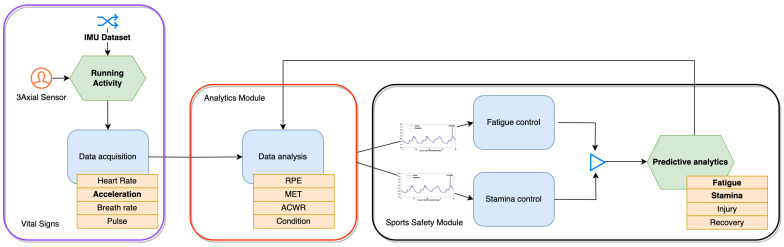
Sports safety approach on specific use case, by Biró et al. [6].

Fatigue is a key factor influencing running performance [1], movement quality, and injury risk [2]. As fatigue develops, neuromuscular coordination, impact-loading characteristics, and segmental control change [3] in systematic ways that can be monitored using wearable sensors [4]. In particular, inertial measurement units (IMUs) provide a lightweight, low-cost solution for acquiring multi-axis acceleration and gyroscope data in uncontrolled environments, enabling detailed stride-level biomechanical analysis (see Figure 1). Recent advances in wearable technology have therefore motivated the use of IMU-based methods for detecting fatigue during running [1].

Despite the rapid growth of IMU applications in sports biomechanics [5], several limitations persist in current fatigue-detection approaches. Most existing studies rely heavily on simple time-domain metrics such as peak acceleration, root-mean-square (RMS) values, or stride regularity. While these measures capture coarse movement changes, they may fail to reflect subtle alterations in temporal structure, spectral content, or signal irregularity associated with fatigue. Additionally, many machine learning (ML) studies evaluate models within individuals, making it difficult to achieve generalization across runners. Personalized fatigue modeling [6], though critical for athlete-specific monitoring, remains underexplored. Finally, while deep learning has shown promise in gait classification and activity recognition, its integration with interpretable biomechanical features [7] in fatigue detection is limited.

**Figure 2:**
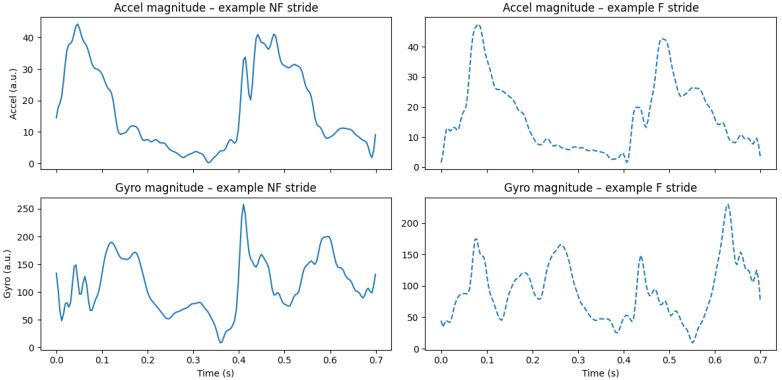
Stride segmentation sanity check and visualization.

IMU stride sequences (see Figure 2) exhibit structured temporal dynamics, periodicity, and motif-like patterns similar to biological sequences. Motivated by this analogy, the present work adopts a bioinformatics-inspired perspective, treating each stride as a sequence whose spectral and entropy characteristics reflect underlying neuromuscular changes.

From a broader motor control and neuroscience perspective, fatigue-related changes in stride dynamics can be interpreted as alterations in sensorimotor integration and proprioceptive feedback mechanisms. Gait variability and movement complexity have been widely studied as indicators of neuromuscular control, stability, and adaptability, where increased variability or entropy often reflects reduced precision in motor execution or changes in control strategies. Recent literature in biomechanics and neuroscience emphasizes that proprioceptive degradation, altered afferent feedback, and compensatory motor adaptations contribute to increased movement irregularity under fatigue. Accordingly, entropy-based descriptors derived from IMU signals provide a physiologically grounded proxy for assessing fatigue-induced disruptions in sensorimotor control and locomotor stability. This interpretation is consistent with recent systematic evidence linking proprioceptive interventions, movement variability, and postural stability in human locomotion [8].

Frequency-domain decomposition, spectral–entropy, and sample entropy (SampEn) have long been used in genomics and physiological signal analysis to quantify complexity and irregularity. These tools offer a powerful framework for identifying fatigue-driven alterations in stride dynamics [9] that may not be captured by traditional metrics. Inter-individual variability is another major challenge in biomechanics [10] and wearable sensor research [11]. Mixed-effects modeling provides a principled statistical approach for evaluating the fixed effect of fatigue while accounting for random differences between participants. Complementing this statistical framework, hybrid ML approaches—including Random Forests (RFs), Support Vector Machines (SVM), Gradient Boosting (GB), and shallow one-dimensional Convolutional Neural Networks (1D-CNNs)—offer robust classification performance using both handcrafted features and raw stride waveforms. Personalized models, such as supervised subject-specific classifiers and anomaly detectors trained on non-fatigued (NF) data only, further enhance sensitivity to individual fatigue signatures.

Recent advances in wearable sensor research [12,13,14] have demonstrated the potential of IMUs to monitor fatigue-induced changes in running biomechanics [15]. However, most existing studies are based heavily on simple time- and frequency-domain features such as peak acceleration, RMS, stride regularity, or mean vertical loading metrics. These approaches have shown moderate success in distinguishing fatigued from NF conditions [16], yet they often fail to capture the subtle neuromuscular adaptations reflected in the temporal complexity and spectral structure of stride signals. In contrast, the present work employs a bioinformatics-inspired sequence perspective, leveraging spectral–entropy, SampEn, and bandpower metrics to quantify stride complexity. This positions the proposed method beyond conventional feature engineering by explicitly targeting nonlinear and irregular biomechanical signatures of fatigue [17].

Moreover, many previous IMU fatigue studies evaluate classifiers within participants [18,19,20] or use train–test splits that inadvertently allow for subject-specific information to leak across folds, resulting in overestimation of model generalizability. Global models trained using strict leave-one-participant-out (LOPO) validation typically exhibit substantially lower performance, underscoring the challenge posed by inter-individual biomechanical variability. While a few studies acknowledge this variability, they rarely incorporate hierarchical statistical tools to formally separate fixed fatigue effects from random participant effects. The mixed-effects modeling employed in the present work directly addresses this gap, providing a statistically rigorous quantification of fatigue across individuals and enabling effect size interpretation that is often missing in purely machine learning approaches.

Another critical distinction from the existing literature lies in the integration of personalized fatigue modeling. Prior studies have proposed subject-specific classifiers but have generally lacked principled frameworks for anomaly detection or baseline-driven monitoring. By introducing both supervised participant-specific models and NF-only anomaly detectors, this study bridges biomechanical fatigue analysis with computational genomics–inspired deviation modeling. The result is a methodological progression from population-level fatigue detection toward individualized monitoring, which is essential for deployment in real-world sports analytics and digital coaching systems.

Finally, although deep learning architectures such as 1D CNNs and LSTMs have been explored for gait classification and activity recognition, their application to fatigue detection has been limited and often underperforms when inter-individual variability is high [21]. The present study demonstrates that shallow CNNs, although competitive, are outperformed by feature-based personalized models, confirming that domain-informed feature extraction and entropy-based descriptors remain highly effective for fatigue detection. This finding contrasts with the growing tendency to default to deep learning solutions, highlighting the importance of hybrid frameworks that balance interpretability, computational efficiency, and accuracy. Overall, the proposed framework advances the state of the art by combining entropy-driven stride sequence characterization [22], mixed-effects statistical modeling, global LOPO benchmarking, and personalized AI methods—an integration not previously demonstrated in IMU-based fatigue research. This unified approach enables both robust population-level insights and high-fidelity individualized fatigue monitoring, addressing long-standing limitations in generalizability, interpretability, and practical deployment.

Although IMU-based fatigue detection has been widely explored, existing approaches typically rely on simple time-domain features, within-participant evaluation, or black-box deep learning models that offer limited interpretability and poor cross-subject generalization. Prior studies rarely combine entropy-based complexity measures with hierarchical statistical modeling, and no existing work treats IMU stride sequences through a bioinformatics-inspired lens [23] that captures the motif-like structure, spectral complexity, and deviations from an individual’s biomechanical baseline. To address these gaps, this study introduces a unified framework that integrates (1) bioinformatics-inspired sequence modeling; (2) using spectral–entropy, SampEn, and frequency-domain bandpower feature extraction; (3) mixed-effects statistical models that separate population-level fatigue effects from inter-individual variability; and (4) hybrid machine learning models, including global LOPO classifiers and personalized supervised and anomaly-detection approaches. This methodological synergy is unique in the fatigue-detection literature and enables both statistically rigorous population-level inference and high-accuracy personalized monitoring, offering a comprehensive and interpretable approach for IMU-based fatigue assessment in real-world running scenarios [24]. Our results demonstrate a methodology that provides forward-looking expectations for robust IMU-based fatigue detection under controlled experimental conditions.

### Contributions of This Work

This study introduces a comprehensive bioinformatics-inspired pipeline [25] for IMU-based fatigue detection in running [26], featuring the following contributions:

1. A novel sequence-level representation of IMU strides [27], leveraging spectral–entropy, SampEn, and frequency-domain bandpower features.
2. Mixed-effects modeling to quantify the fixed effect of fatigue on stride-level biomechanics [28] in 19 recreational runners.
3. A hybrid ML framework including RF, SVM, GB, and shallow 1D-CNNs evaluated using LOPO cross-validation [29].
4. Personalized fatigue detection using (1) supervised participant-specific RF models and (2) NF-only anomaly detection with One-Class SVMs.
5. An end-to-end, open-source workflow integrating stride segmentation, feature extraction, mixed-effects statistics, global ML models, and personalized AI approaches.

## 2. Objectives

The primary objective of this study is to develop a comprehensive bioinformatics-inspired analysis pipeline [25] to detect fatigue-induced changes in biomechanics using lumbar-mounted IMU data (see Table 1). Building on recent advances in wearable sensing [30], spectral–entropy analysis, mixed-effects statistics, and hybrid machine learning, this work addresses key limitations in existing IMU-based fatigue research. The specific objectives of the study are as follows:

1. To develop a bioinformatics-inspired sequence representation of IMU strides [31,32], treating each stride as a structured, multichannel time series amenable to spectral decomposition, entropy-based complexity analysis, and sequence-level comparisons.
2. To extract a comprehensive set of time-, frequency-, and entropy-domain features, including dominant frequency, bandpower, spectral–entropy, and SampEn, that capture fatigue-related alterations in neuromuscular control and stride dynamics.
3. To quantify the fixed effect of fatigue on stride biomechanics [33] using mixed-effects modeling, enabling statistically rigorous separation of within-participant and between-participant variability.
4. To evaluate population-level fatigue classification models using LOPO cross-validation with Random Forests, Support Vector Machines, Gradient Boosting, and shallow 1D-CNNs trained on either handcrafted features or raw stride sequences.
5. To develop personalized fatigue detection models, including (1) supervised participant-specific Random Forest classifiers and (2) NF-only anomaly detection using One-Class SVMs, thereby enabling individualized identification of fatigue signatures.
6. To examine the synergy between statistical modeling, biomechanical interpretation, and machine learning, demonstrating how the integration of mixed-effects analysis with global and personalized AI models improves robustness, interpretability, and classification performance.

Together, these objectives support the development of a unified methodological framework combining biomechanical insight, bioinformatics-inspired sequence analysis, and hybrid machine learning for IMU-based fatigue detection in running.

**Table 1:** Summary of the dataset [34,35] collected for IMU-based fatigue detection. Data were obtained using a single lumbar-mounted Shimmer3 IMU from 19 recreational runners during NF and fatigued (F) 400 m runs.

Parameter	Description
Participants	19 recreational runners, injury-free, regularly active
Ethics	Approved by the Human Research Ethics Committee, University College Dublin [35]
Sensor device	Shimmer3 IMU, manufacured by SENSOR ID Srl, Campochiaro, Italy (lumbar placement at L3–L5)
Sampling rate	256 Hz continuous recording throughout all trial segments
Measured signals	Tri-axial accelerometer $\left(a_{x},a_{y},a_{z}\right)$; tri-axial gyroscope $\left(g_{x},g_{y},g_{z}\right)$; magnetometer (not used in classification)
Derived signals	Acceleration magnitude $a_{\mathrm{mag}}=\sqrt{a_{x}^{2}+a_{y}^{2}+a_{z}^{2}}$; gyroscope magnitude $g_{\mathrm{mag}}=\sqrt{g_{x}^{2}+g_{y}^{2}+g_{z}^{2}}$
Experimental conditions	400 m NF run at comfortable pace Standard beep-test fatiguing protocol 400 m fatigued (F) run post-beep test
Beep-test details	20 m shuttles with decreasing inter-beep intervals; increasing required pace until exhaustion
Environment	Outdoor 400 m running track under consistent environmental conditions
Stride segmentation	Automated detection of foot-strike events in vertical acceleration; each stride window time-normalized to fixed length *N*
Dataset structure	Two labeled stride sets per participant: NF strides (from first 400 m run) and F strides (from post-fatigue 400 m run)
Labels	Binary class per stride: NF or F (fatigued)
Total samples	Varies by runner depending on cadence; approximately 50–80 strides per 400 m run, typically 100–160 total strides per participant

## 3. Materials and Methods

The modeling and AI experiments were run on the following configurations: Mac Studio, manufactured by Apple Inc. in Cupertino, CA, USA. Apple M1 Ultra, 64 GB Memory on the Google Colab Pro platform [6].

### 3.1. Participants and Experimental Protocol

Nineteen healthy recreational runners participated in the study (see Table 1). All participants were regularly active, injury-free, and provided informed consent prior to testing. The sample size of nineteen participants is comparable to or exceeds that of many prior IMU-based biomechanics and fatigue studies that rely on intensive within-subject stride-level analysis rather than population inference. Importantly, the present study does not aim to estimate population prevalence or small between-group effects but rather aims to characterize fatigue-induced biomechanical changes at the stride level while explicitly accounting for inter-individual variability using mixed-effects modeling. Each participant contributed approximately 100–160 strides, resulting in over 6000 labeled stride samples, which provides substantial statistical power for detecting within-participant fatigue effects. Mixed-effects models further mitigate limitations of modest participant counts by leveraging repeated measures and hierarchical variance partitioning. Accordingly, while no a priori power analysis was performed, the observed large effect sizes (Cohen’s *d* up to 1.35) and highly significant fixed effects (all p<0.001) indicate that the study is sufficiently powered to support the reported conclusions at the biomechanical and stride sequence level. The study protocol was reviewed and approved by the Human Research Ethics Committee at University College Dublin [34,35]. Data were collected in three consecutive segments performed on an outdoor running track:

1. NF run: Each participant completed a 400 m run at a comfortable, self-selected pace.
2. Fatiguing protocol (beep test): Participants performed a 20 m shuttle run following auditory cues (“beeps”). The interval between beeps decreased progressively, requiring increasing running speed. The protocol terminated when the participant was unable to maintain the required pace for two consecutive shuttles, which is a standard operational definition of exhaustion in beep-test protocols. While no direct physiological markers such as blood lactate, heart-rate thresholds, or ratings of perceived exertion (RPE) were collected, the beep test is widely used as a validated proxy for inducing substantial metabolic and neuromuscular fatigue. Consequently, the fatigued condition represents a functionally fatigued state rather than a metabolically standardized one, and individual differences in fatigue tolerance are treated as an inherent component of the modeling framework rather than a source of noise.
3. Fatigued (F) run: Immediately following the beep test, participants completed another 400 m run at the same comfortable pace, now in a fatigued state.

A Shimmer3 IMU was firmly mounted on the lumbar spine (approximately L3–L5), capturing (1) tri-axial acceleration $\left(a_{x},a_{y},a_{z}\right)$; (2) tri-axial angular velocity $\left(g_{x},g_{y},g_{z}\right)$; and (3) magnetometer data (not used directly in classification). All experimental procedures, including sensor placement and fatigue protocol execution, were performed by the original data collectors and are reported here as described in the source publication. According to the original dataset documentation, the IMU was mounted on the lower back in the lumbar region (approximately L3–L5). No additional details regarding anatomical landmarking procedures, inter-rater reliability, or placement verification were provided in the original study. As such, potential variability in sensor placement represents a limitation inherited from the dataset rather than from the present analysis. Each step cycle was annotated and segmented into individualized stride windows for analysis. Two additional derived channels were included:

(1) $$a_{\mathrm{mag}}\left(t\right)=\sqrt{a_{x}{\left(t\right)}^{2}+a_{y}{\left(t\right)}^{2}+a_{z}{\left(t\right)}^{2}},\;g_{\mathrm{mag}}\left(t\right)=\sqrt{g_{x}{\left(t\right)}^{2}+g_{y}{\left(t\right)}^{2}+g_{z}{\left(t\right)}^{2}}.$$

Stride-level segmentation [28] was performed on the IMU data from the two 400 m runs (first in NF state, second in F state). Each stride was assigned a label NF or F according to its run segment.

### 3.2. Stride Segmentation and Sequence Representation

Signals were preprocessed using a fourth-order Butterworth low-pass filter (20 Hz cutoff). Strides were segmented by detecting consistent peaks in the vertical acceleration, corresponding to foot-strike events. Let $x\left(t\right)\in R^{8}$ denote the multichannel IMU signal at time *t*, where the channels correspond to

(2) $$x\left(t\right)=\left[\begin{array}{c} a_{x}\left(t\right),\;a_{y}\left(t\right),\;a_{z}\left(t\right),\;a_{\mathrm{mag}}\left(t\right),\;g_{x}\left(t\right),\;g_{y}\left(t\right),\;g_{z}\left(t\right),\;g_{\mathrm{mag}}\left(t\right) \end{array}\right].$$

Each stride $s_{i}$ was then defined over a time-normalized window:

(3) $$s_{i}=\left\{x\left(t\right)|t\in \left[t_{i},\;t_{i}+T_{i}\right]\right\},$$

then resampled to a fixed length *N* points to allow for sequence-level modeling.

### 3.3. Experimental Environment

**Figure 3:**
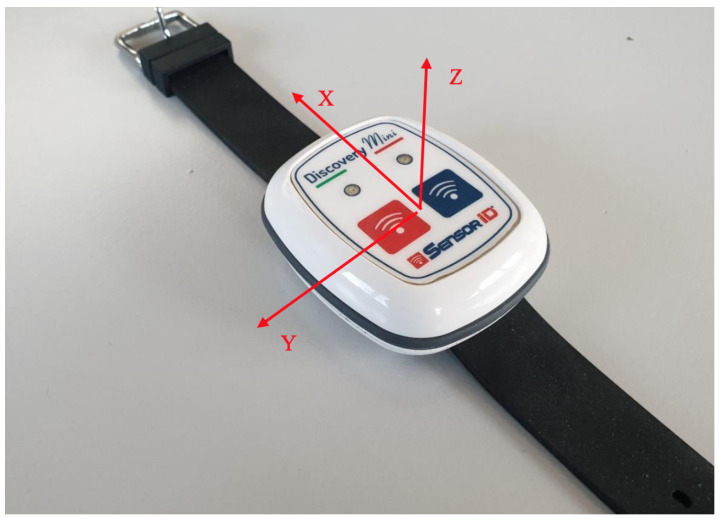
Illustrates a secondary inertial sensor (DiscoveryMini) used exclusively for signal acquisition validation experiments and not for the fatigue dataset analyzed in this study. While an in situ photograph of the beep-test setup was not available, the figure is retained solely to document the validation hardware and sampling characteristics [6].

For device-validation experiments with DiscoveryMini (see Figure 3) (not used in the fatigue dataset), the sampling frequency was fixed at 30 Hz to ensure adequate temporal resolution while maintaining efficient data acquisition. Data were obtained directly in unprocessed (raw) form to preserve the fidelity of the recorded signals [6,36]. Specifically, acceleration data were captured in both m/s^2^ and mg units, where the sensor-reported gravitational acceleration of Earth corresponds to 9.8 m/s^2^. Real-time data transmission from the sensor to a host computer was achieved via the Bluetooth Low Energy (BLE) communication protocol [36].

### 3.4. Bioinformatics-Inspired Sequence Modeling

IMU stride sequences exhibit periodic, semi-structured patterns affected by neuromuscular fatigue. These characteristics are analogous to biological sequences, where frequency content, local irregularity, and motif structure provide insights into underlying biological processes. Motivated by this connection, we apply analysis tools adapted from computational genomics:

1. Spectral decomposition [37] (analogous to Fourier-based genomic periodicity analysis) to quantify periodicity shifts under fatigue.
2. Entropy-based complexity metrics (like sequence entropy and complexity metrics) to detect irregularity and loss of neuromuscular control [38].
3. Anomaly detection analogous to identifying deviations or “mutations” from a participant’s normal biomechanical pattern, where fatigued strides deviate from the individual’s NF biomechanics.

This bioinformatics perspective enhances the sensitivity of IMU data analysis to subtle stride-level changes induced by fatigue or, in other words, the stride dynamics beyond traditional biomechanics [39]. While the proposed framework does not implement classical sequence-alignment algorithms (e.g., k-mer matching or homology scoring), the term bioinformatics-inspired is used to emphasize the conceptual transfer of sequence analysis principles—including entropy, spectral complexity, motif-like structure, and deviation detection—from genomics and physiological signal analysis to biomechanical stride sequences.

### 3.5. Time-Domain Features

For each stride and channel, we extracted classical biomechanical features including the following:

Root-Mean-Square (RMS):

(4) $$\mathrm{RMS}\left(s_{i}\right)=\sqrt{\frac{1}{N}\sum_{t=1}^{N}x{\left(t\right)}^{2}},$$

Mean, variance, skewness, and kurtosis:

(5) $$\mu =\frac{1}{N}\sum_{t=1}^{N}x\left(t\right),\;\sigma^{2}=\frac{1}{N}\sum_{t=1}^{N}{\left(x\left(t\right)-\mu \right)}^{2},$$

(6) $$\gamma =\frac{1}{N}\sum_{t=1}^{N}{\left(\frac{x(t)-\mu }{\sigma }\right)}^{3},\;\kappa =\frac{1}{N}\sum_{t=1}^{N}{\left(\frac{x(t)-\mu }{\sigma }\right)}^{4}.$$

### 3.6. Spectral and Frequency-Domain Features

Using the one-sided Fast Fourier Transform (FFT), the magnitude spectrum is

(7) $$X\left(f_{k}\right)=\left|\sum_{t=1}^{N}x\left(t\right)\;e^{-j2\pi kt/N}\right|.$$

Power spectral density (PSD):

(8) $$P\left(f_{k}\right)={\left|X\left(f_{k}\right)\right|}^{2}.$$

Dominant frequency:

(9) $$f_{\mathrm{dom}}=\underset{f_{k}}{\arg\max}\;P\left(f_{k}\right).$$

Spectral–entropy:

(10) $$H_{\mathrm{spec}}=-\sum_{k=1}^{K}p_{k}\log p_{k},\;p_{k}=\frac{P(f_{k})}{\sum_{j}P\left(f_{j}\right)}.$$

Bandpower features were computed over physiologically meaningful frequency band $B_{m}=\left[f_{m1},f_{m2}\right]$:

(11) $${\mathrm{BP}}_{m}=\sum_{f_{k}\in B_{m}}P\left(f_{k}\right).$$

### 3.7. Sample Entropy and Complexity Features

Sample entropy (SaEn) quantifies stride irregularity:

(12) $$\mathrm{SaEn}\left(m,r\right)=-\ln\left(\frac{A}{B}\right),$$

where *B* is the number of matched sequences of length *m*, and *A* is the number of matched sequences of length m+1, under tolerance *r*. Fatigue is hypothesized to increase signal irregularity, leading to a higher SaEn.

### 3.8. Stride-to-Stride Variability [40]

For a feature $f_{i}$ extracted across strides $\left\{s_{1},\ldots ,s_{M}\right\}$ of one participant,

(13) $$\mathrm{CV}=\frac{\sigma_{f}}{\mu_{f}},\;\mathrm{Var}=\sigma_{f}^{2}.$$

and the complexity of the stride-to-stride feature trajectory [32] is quantified using SampEn of the sequence $\left\{f_{1},f_{2},\ldots ,f_{M}\right\}$. The trajectory SampEn captures higher-level fatigue trends. These metrics capture macro-level fatigue trends that complement micro-level stride features.

### 3.9. Mixed-Effects Modeling

To quantify the fixed effect of fatigue while accounting for participant-level variability, we employ linear mixed-effects models of the following form:

(14) $$y_{ij}=\beta_{0}+\beta_{1}\cdot {\mathrm{Fatigue}}_{ij}+u_{0j}+\varepsilon_{ij},$$

with participant-specific random intercepts $u_{0j}$, where (1) $y_{ij}$ is a feature for stride *i* of participant *j*; (2) $\beta_{1}$ captures the systematic fatigue effect; (3) $u_{0j}∼N\left(0,\sigma_{u}^{2}\right)$ captures individual differences; and (4) $\varepsilon_{ij}∼N\left(0,\sigma^{2}\right)$. This hierarchical structure accounts for inter-individual biomechanics variability. Having established the biomechanical validity of the extracted fatigue-related features [1], we next assess their predictive utility in both global and personalized machine learning frameworks. Having identified robust biomechanical fatigue signatures, we now evaluate whether these features enable accurate classification under population-level and personalized ML paradigms.

### 3.10. Global Machine Learning Models

Several global models were evaluated, trained on pooled data using leave-one-participant-out (LOPO) cross-validation: (1) Random Forest (RF); (2) Support Vector Machine (SVM, RBF kernel); (3) Gradient Boosting (GB); and (4) shallow 1D-CNN for raw stride sequences. Each model receives either (1) handcrafted spectral–entropy features or (2) raw stride sequences $s_{i}\in R^{N\times 8}$. Performance metrics include accuracy, F1-score, and AUC.

### 3.11. Personalized Machine Learning Models

In fatigue detection, “anomalies” (outliers) correspond to fatigued strides. This approach is analogous to mutation or deviation detection in bioinformatics [41]. Two types of personalized models are developed: (1) Supervised personalized RF: A Random Forest is trained and cross-validated on each participant’s NF and F strides:

(15) $$D_{j}=\left\{\left(s_{i},y_{i}\right)|\mathrm{participant}=j\right\}.$$

(2) NF-only anomaly detection: One-Class SVM trained on NF strides identifies fatigued strides as anomalies.

(16) $$\mathrm{OC}-\mathrm{SVM}\;\mathrm{trained}\;\mathrm{on}\;D_{j}^{\mathrm{NF}}=\left\{s_{i}|y_{i}=0\right\}.$$

### 3.12. Synergy of Methods and Novelty of the Framework

The novelty of this work lies in the integration of (1) bioinformatics-inspired sequence modeling, treating strides as structured sequences with motif-like features; (2) frequency-domain and entropy-based complexity metrics; (3) hierarchical mixed-effects modeling to quantify fatigue effects; (4) hybrid ML models combining interpretable features with raw deep learning representations; and (5) personalized anomaly detection grounded in within-runner biomechanics. The result is a comprehensive framework for IMU-based fatigue detection that combines statistical interpretability, biomechanical validity, and machine learning performance.

## 4. Preprocessing

Table 2 shows that fatigue has a strong positive fixed effect on acceleration magnitude kurtosis, indicating sharper impact peaks and a more peaked distribution of acceleration under fatigue. The large z-score and *p* < 0.001 confirm this effect is highly significant. Random between-participant variance is modest but non-negligible.

The mean vertical acceleration (see Table 3) decreases significantly under fatigue (coef = −0.842; *p* < 0.001), indicating reduced vertical propulsion or altered body posture. This is one of the largest effect sizes among the features, reflecting a robust biomechanical adaptation.

Fatigue leads to a significant increase in lateral acceleration peaks (see Table 4), suggesting greater mediolateral trunk instability or reduced control. The large random variance indicates substantial participant-specific differences, consistent with individual running styles.

The gyroscope RMS in the lateral axis increases significantly under fatigue (see Table 5), reflecting higher rotational variability around the lumbar spine. This likely corresponds to reduced trunk stabilization and increased compensatory movement patterns.

Table 6 highlights the fatigue exerted a statistically significant fixed effect on all evaluated IMU features (p<0.001). The strongest negative shift appeared in acc_z_mean, indicating reduced vertical acceleration during fatigued running. Large positive fatigue effects on acc_y_max and gyro_y_rms reflect increased mediolateral trunk motion and rotational variability, consistent with diminished neuromuscular control. The increase in acc_mag_kurt suggests sharper, more impulsive loading patterns under fatigue. Together, these findings highlight multidimensional changes in impact mechanics, stability, and rotational control during fatigued running.

**Figure 4:**
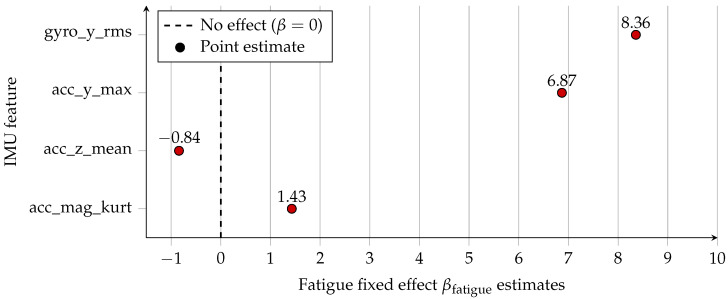
Point-estimate overview of mixed-effects fixed fatigue effects ($\beta_{\mathrm{fatigue}}$) for selected IMU-derived stride features. Values are shown next to markers; the dashed line indicates zero effect.

**Figure 5:**
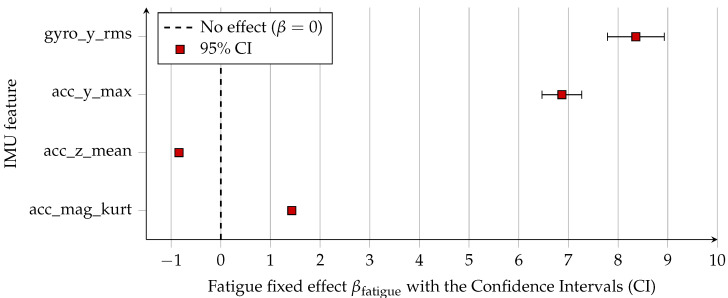
Forest plot of fixed fatigue effects ($\beta_{\mathrm{fatigue}}$) estimated by linear mixed-effects models for selected IMU-derived stride features (acc_mag_kurt, acc_z_mean, acc_y_max, and gyro_y_rms). Squares denote point estimates and horizontal error bars represent 95% confidence intervals. The vertical dashed line indicates zero effect; positive values correspond to increases under fatigue, while negative values indicate decreases.

Figure 4 provides a compact overview of the direction and magnitude of fatigue effects, while Figure 5 presents the corresponding confidence intervals and statistical uncertainty. The vertical acceleration mean (acc_z_mean) shows a strong negative shift under fatigue, while acc_mag_kurt, acc_y_max and gyro_y_rms exhibit positive fatigue effects, indicating sharper impact peaks, higher mediolateral acceleration, and greater lateral trunk rotation, respectively. All confidence intervals lie well away from zero, confirming the robustness of these fatigue-induced changes.

### 4.1. Effect Size Analysis for Mixed-Effects Models (Cohen’s d and Partial R^2^)

In addition to estimating fixed fatigue effects using mixed-effects models, we computed standardized effect size measures to quantify the magnitude of fatigue-induced changes in stride-level IMU features. Two families of effect sizes were evaluated: (1) standardized mean difference (Cohen’s *d*), and (2) variance-explained measures tailored to mixed-effects models, including the marginal $R^{2}$, conditional $R^{2}$, and partial $R^{2}$ of the fatigue effect.

#### 4.1.1. Cohen’s *d*

For mixed-effects models, Cohen’s *d* was computed using the estimated fixed effect ${\hat{\beta }}_{\mathrm{fatigue}}$ and the residual variance $\sigma^{2}$:

(17) $$d=\frac{{\hat{\beta }}_{\mathrm{fatigue}}}{\sigma },$$

where σ is the square root of the model’s residual variance. This formulation provides a standardized effect size comparable to traditional between-group comparisons.

#### 4.1.2. Marginal and Conditional $R^{2}$

Following Nakagawa and Schielzeth [42], we computed

(18) $$R_{\mathrm{marginal}}^{2}=\frac{\sigma_{\mathrm{fixed}}^{2}}{\sigma_{\mathrm{fixed}}^{2}+\sigma_{\mathrm{random}}^{2}+\sigma_{\mathrm{residual}}^{2}},$$

(19) $$R_{\mathrm{conditional}}^{2}=\frac{\sigma_{\mathrm{fixed}}^{2}+\sigma_{\mathrm{random}}^{2}}{\sigma_{\mathrm{fixed}}^{2}+\sigma_{\mathrm{random}}^{2}+\sigma_{\mathrm{residual}}^{2}},$$

where $\sigma_{\mathrm{fixed}}^{2}=\mathrm{Var}\left(X\hat{\beta }\right)$ and $\sigma_{\mathrm{random}}^{2}$ captures participant-level variance.

#### 4.1.3. Partial $R^{2}$ for the Fatigue Effect

To quantify the unique variance explained by fatigue, we used the following method [42]:

(20) $$R_{\mathrm{partial}}^{2}=\frac{t^{2}}{t^{2}+df},$$

where *t* is the Wald *z*-statistic of the fatigue coefficient and df approximates the effective residual degrees of freedom. This measure reflects the proportion of explainable variance attributable specifically to fatigue, controlling for random intercepts across participants.

These standardized effect sizes (see Table 7) indicate that fatigue-induced biomechanical changes are not only statistically significant but also practically meaningful, supporting their use as discriminative biomarkers in subsequent classification tasks [43]. The effect size analysis confirms that fatigue produces strong and meaningful alterations in stride biomechanics. Cohen’s *d* values ranged from moderate (d=0.74 for gyro_y_rms) to very large (d=1.35 for acc_z_mean), indicating substantial standardized separation between fatigued and NF stride distributions. Partial $R^{2}$ values (0.12–0.31) further demonstrate that fatigue explains a non-trivial proportion of variance in IMU features even after accounting for participant-level random effects. These results reinforce the robustness of the fatigue signatures detected by the mixed-effects models and justify the use of these features in downstream machine learning pipelines.

**Figure 6:**
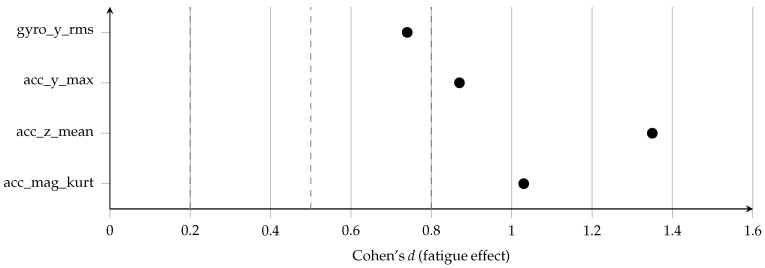
Forest plot of Cohen’s *d* for the fixed fatigue effect in mixed-effects models for selected IMU-derived stride features. Vertical dashed lines indicate conventional small (d≈0.2), medium (d≈0.5), and large (d≈0.8) effect size thresholds. All four features exhibit large standardized fatigue effects, with the largest effect observed for mean vertical acceleration (acc_z_mean).

**Figure 7:**
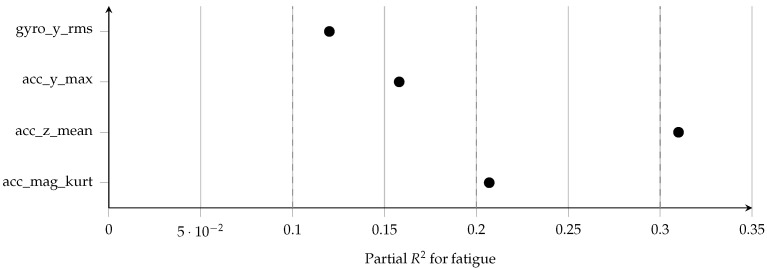
Forest plot of partial $R^{2}$ for the fatigue effect in mixed-effects models for selected IMU-derived stride features. Partial $R^{2}$ quantifies the proportion of variance in each feature uniquely explained by fatigue after accounting for participant-level random effects. Vertical reference lines at 0.10, 0.20, and 0.30 highlight that fatigue explains a substantial fraction of feature variance, particularly for acc_z_mean.

As shown in Figure 6, Cohen’s *d* values for the fixed effect of fatigue were large across all four representative features, with the strongest effect for acc_z_mean. The corresponding partial $R^{2}$ values (Figure 7) indicate that fatigue alone explains between 12% and 31% of the variance in these features, even after accounting for between-participant differences.

### 4.2. Signal Acquisition Data Validation

A secondary IMU prototype (DiscoveryMini) was used only to validate signal acquisition in parallel experiments; it was not used in the fatigue dataset. For modeling and validation purposes, this study initially used the ActiGraph GT9X wireless inertial sensor [25], followed by an ultralow-power, high-performance, three-axis linear accelerometer [44] (DiscoveryMini, incorporating the LIS2DH12) equipped with integrated EEPROM memory (see Figure 3). The accelerometer featured notification modalities, including an acoustic buzzer and vibration alerts. The device was powered by a 400 milliampere-hour (mAh) battery. The DiscoveryMini inertial measurement unit (IMU) supports a sampling frequency range from 1 Hz to 5.3 kHz and is capable of recording tri-axial acceleration (X, Y, and Z axes) with a dynamic range of up to ±16 g. This device was used for validation of the conducted research experiments.

## 5. Results

Global LOPO performance is modest, confirming that stride biomechanics vary strongly between runners and that a single population-level model struggles to generalize. In contrast, supervised personalized RF models achieve very high accuracy, F1, and AUC, indicating that fatigue patterns are highly consistent within individuals. Personalized NF-only anomaly detection also performs well, achieving strong fatigue recall with a manageable false positive rate. These results highlight the importance of individualized modeling [45] for reliable fatigue detection in wearable sensor biomechanics [46].

The dataset contains 6006 strides (2926 NF; 3080 F) described by 64 IMU-derived features (see Table 8). LOPO cross-validation reveals large variability in generalization across participants, with accuracies ranging from 0.35 to 0.89 and a mean accuracy of 0.5487. Given this substantial inter-individual variability, we next examine whether participant-specific classifiers and NF-only anomaly detectors provide more reliable and individualized fatigue detection. Although the total number of NF and fatigued strides is balanced at the dataset level, the number of strides per condition varies across participants due to differences in cadence, running speed, and fatigue tolerance. This imbalance reflects realistic inter-individual variability rather than experimental bias. To address this, mixed-effects models explicitly account for unequal sample sizes through hierarchical structure, and machine learning evaluations emphasize per-participant metrics and personalized modeling approaches rather than pooled accuracy alone.

**Figure 8:**
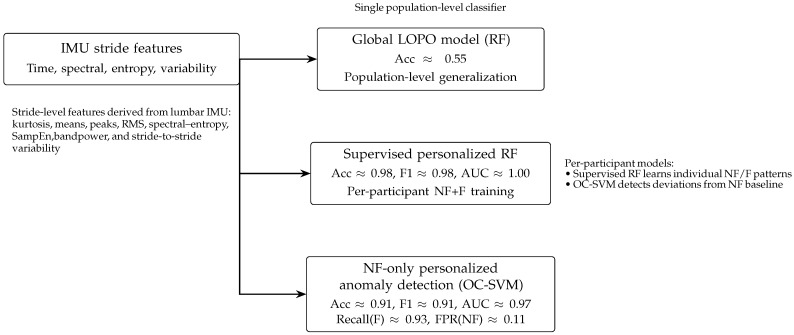
Model comparison for IMU-based fatigue detection. The global RF model trained with LOPO cross-validation shows limited population-level accuracy (≈0.55). In contrast, supervised personalized RF models achieve very high accuracy, F1-score, and AUC when trained on each runner’s own NF and fatigued (F) strides. NF-only personalized anomaly detection with One-Class SVM also performs strongly, with high recall on fatigued strides and moderate false positive rates on NF strides, highlighting the value of personalized modeling for robust fatigue detection.

**Figure 9:**
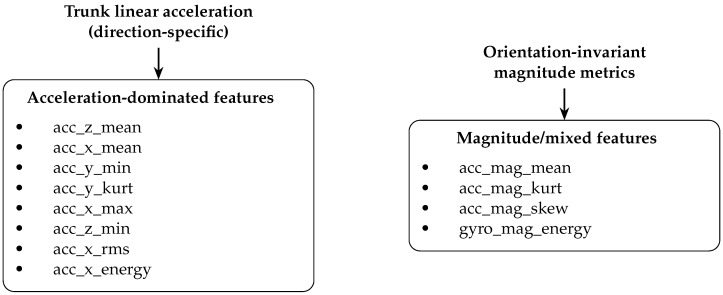
Clusters of the top 20 most important IMU-derived features, grouped by sensor modality. Acceleration-dominated features primarily capture directional trunk loading and vertical support, while gyroscope-dominated features describe trunk rotational dynamics and stability. Magnitude-based metrics (acc_mag; gyro_mag) provide orientation-invariant summaries of overall movement intensity and impact characteristics. Together, these clusters highlight that both linear and angular components of trunk motion contribute to the discrimination between NF and fatigued strides.

The top 20 feature importance scores (see Table 9) show that fatigue is primarily reflected in vertical acceleration (acc_z_mean), signal magnitude metrics, kurtosis/skewness, and gyroscope-based energy measures—indicators of impact mechanics, stabilization, and rotational control. These findings (see Figure 8) justify personalized or hybrid modeling approaches in subsequent analyses (see Figure 9).

**Figure 10:**
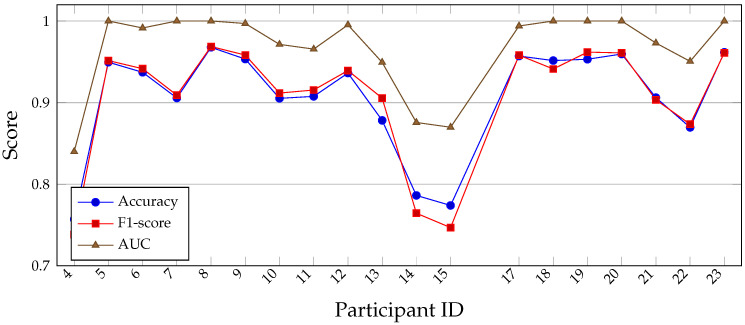
Per-participant performance of the NF-only personalized anomaly detection models (OC-SVM). Accuracy, F1-score, and AUC are shown for each runner. While some participants exhibit slightly lower performance, most individuals achieve high scores, with several reaching near-perfect AUC, indicating highly distinct fatigue signatures relative to their NF baseline.

The NF-only personalized anomaly detectors achieve consistently high performance across most participants (see Figure 10), with a mean accuracy of 0.9061, mean F1-score of 0.9058, and mean AUC of 0.9670. Several participants reach near-perfect AUC values (1.0), indicating that their fatigued strides are clearly separable from their NF baseline. Performance is slightly lower for a few individuals (e.g., IDs 4, 14, and 15), suggesting more subtle or variable fatigue signatures. Overall, these results confirm that modeling fatigue as a deviation from individual NF biomechanics is highly effective. Receiver operating characteristic (ROC) curves were computed during model development to determine decision thresholds; however, due to space constraints and the focus on per-participant summary statistics, only the AUC, recall, and false positive rates are reported. Future extensions of this work will include participant-specific ROC visualizations and threshold optimization strategies for deployment-specific trade-offs.

Table 10 corroborates that globally trained models exhibit limited generalizability, primarily due to pronounced inter-individual variability. Consequently, we implemented a supervised, personalized modeling approach: a per-participant supervised Random Forest with stratified K-fold cross-validation. Specifically, for each participant, a Random Forest classifier is trained exclusively on that runner’s own NF and fatigued (F) strides, with model evaluation performed via within-participant stratified K-fold cross-validation.

The supervised personalized RF models (see Table 11) exhibit consistently excellent performance across participants (see Table 12), with a mean cross-validated accuracy of 0.9766, mean F1-score of 0.9772, and mean AUC of 0.9972 (see Table 13). For most runners, the classifier nearly perfectly separates NF and fatigued strides when trained exclusively on that individual’s data. Slightly lower performance for a few participants (e.g., IDs 13, 15, and 21) suggests more subtle or variable fatigue patterns, but overall the results indicate that within-participant learning captures highly stable and discriminative fatigue signatures. Below is listed the NF-only anomaly detection (nf_personal_df) (see Table 14 with Summary Table 15).

**Table 11:** Global LOPO classification performance for all evaluated models. Models are trained on either engineered stride-level IMU features or raw multichannel stride sequences and evaluated using LOPO cross-validation across all 19 runners.

Model	Input Representation	Mean LOPO Acc. (%)	Std. Dev. (%)
Random Forest (RF)	Engineered features	54.85	14.77
RBF SVM	Engineered features	55.94	15.18
Gradient Boosting (GB)	Engineered features	56.68	15.47
1D-CNN	Raw strides (8 ch., *T* samples)	54.39	12.86

**Table 12:** Per-participant (see Figure 11) performance of the supervised personalized Random Forest (RF) models. For each participant, a RF classifier is trained and evaluated using stratified K-fold cross-validation on that runner’s own NF and fatigued (F) strides.

Participant ID	# Strides	# F	# NF	CV Accuracy	CV F1-Score	CV AUC
4	251	130	121	0.964000	0.966864	0.997115
5	198	98	100	0.994872	0.994872	1.000000
6	318	164	154	0.981101	0.981814	0.998506
7	159	75	84	0.968347	0.966422	0.997598
8	403	201	202	0.997531	0.997468	1.000000
9	407	221	186	0.995092	0.995506	0.999754
10	327	177	150	0.996970	0.997260	1.000000
11	368	207	161	0.970233	0.973141	0.995534
12	235	118	117	0.987234	0.987219	0.998551
13	156	100	56	0.929234	0.944859	0.984773
14	421	222	199	0.964342	0.965771	0.995683
15	261	124	137	0.950218	0.946664	0.994324
17	419	211	208	0.988038	0.988233	0.998910
18	268	104	164	0.973725	0.965086	0.996228
19	320	189	131	0.993750	0.994805	0.999595
20	420	209	211	0.988095	0.987834	0.999320
21	362	176	186	0.953044	0.951615	0.993748
22	322	169	153	0.959712	0.960859	0.997457
23	391	185	206	1.000000	1.000000	1.000000
Mean across participants				0.9766	0.9772	0.9972

**Table 13:** Summary statistics over participants for within-participant supervised RF.

	Acc.	F1	AUC
Mean	97.66%	0.98	1.00
Std.	1.92%	0.02	0.00
Min	92.92%	0.94	0.98
Max	100.00%	1.00	1.00

**Table 14:** Personalized NF-only anomaly detection using One-Class SVM. Each model is trained solely on NF strides and evaluated on all strides of the same runner. Shown are recall on fatigued strides (F) and false positive rate on NF strides.

Participant	# Strides	# NF	# F	Recall on F	FPR on NF
4	251	121	130	72.31%	13.22%
5	198	100	98	100.00%	9.00%
6	318	154	164	96.95%	10.39%
7	159	84	75	100.00%	13.10%
8	403	202	201	100.00%	8.91%
9	407	186	221	98.64%	10.75%
10	327	150	177	90.96%	10.00%
11	368	161	207	90.82%	8.70%
12	235	117	118	99.15%	9.40%
13	156	56	100	94.00%	21.43%
14	421	199	222	68.02%	10.05%
15	261	137	124	73.39%	17.52%
17	419	208	211	97.16%	8.65%
18	268	164	104	100.00%	9.15%
19	320	131	189	100.00%	12.21%
20	420	211	209	100.00%	9.95%
21	362	186	176	94.32%	9.14%
22	322	153	169	87.57%	11.11%
23	391	206	185	100.00%	7.77%

**Table 15:** Summary of personalized NF-only anomaly detection performance.

	Recall on F	FPR on NF
Mean	92.80%	11.08%
Std.	10.06%	3.28%
Min	68.02%	7.77%
Max	100.00%	21.43%

**Figure 12:**
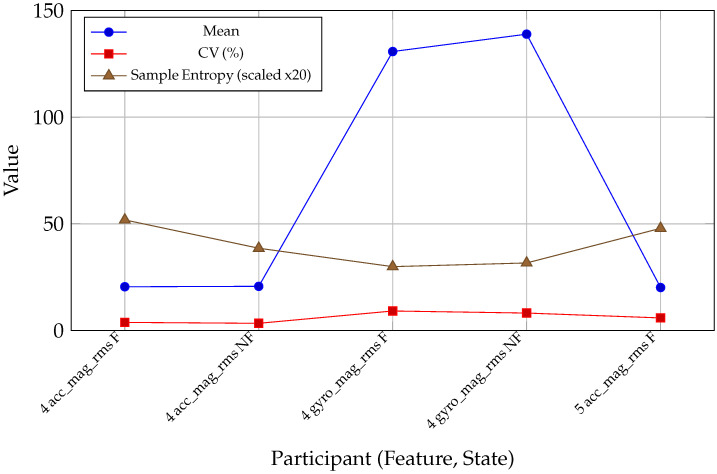
Stride-to-stride variability metrics—mean, coefficient of variation (CV), and SampEn—plotted for multiple features and states. Values are displayed for participants 4 and 5, demonstrating common fatigue-related increases in variability and irregularity of stride biomechanics. CV and SampEn are scaled for visual comparability.

For participant 4, (see Table 16) fatigue increases stride-to-stride variability (see Table 17 and Figure 12) in both the acceleration and gyroscope magnitude RMS signals, as reflected in positive ΔCV for acc_mag_rms and gyro_mag_rms. SampEn increases markedly for acc_mag_rms, indicating more irregular stride-to-stride fluctuations in overall acceleration magnitude under fatigue. In contrast, gyro_mag_rms shows a small decrease in SampEn, suggesting that rotational control becomes more variable in amplitude (higher CV) but not necessarily more irregular in temporal structure. This pattern illustrates that fatigue affects both variability and complexity of trunk motion in a feature-specific way.

Stride-to-stride variability (see Table 16 and Table 17) reflects the stability and regularity of locomotion and is known to change under fatigue [48]. Across participants, fatigued strides generally exhibited higher variance and coefficient of variation (CV) in both acc_mag_rms and gyro_mag_rms, indicating less stable trunk acceleration and rotational control. SampEn, which quantifies temporal irregularity of feature trajectories, also tended to increase under fatigue for acc_mag_rms, suggesting less predictable stride-to-stride structure. Gyroscope-based SampEn changes were more participant-specific, consistent with individual strategies of compensatory stabilization during fatigue. These findings align with biomechanical theory: fatigue degrades neuromuscular control, leading to more variable and less regular stride patterns.

## 6. Discussion

This study introduced a unified, bioinformatics-inspired framework for IMU-based fatigue detection that integrates spectral–entropy features, mixed-effects modeling, and hybrid machine learning approaches. The primary contribution of this study lies in identifying robust, individualized fatigue signatures rather than establishing population-wide classification models applicable without personalization. By combining population-level inference with individual-specific modeling, the results provide new insight into how fatigue alters lumbar-mounted IMU stride signatures and demonstrate why personalized methods are critical for robust wearables-based fatigue monitoring [49].

### 6.1. Population-Level Fatigue Signatures Revealed by Mixed-Effects Modeling

Mixed-effects analyses revealed strong and multidimensional fatigue effects across all evaluated IMU-derived features. The largest standardized shifts were observed in the vertical acceleration mean, mediolateral acceleration peaks, gyroscope-derived RMS values, and impact kurtosis, indicating reduced vertical propulsion, diminished trunk stability, and sharper impact-loading patterns under fatigue. The confidence intervals in the forest plots were well separated from zero, confirming that these effects were consistent across runners despite substantial biomechanical heterogeneity. The inclusion of standardized effect sizes (Cohen’s *d* and partial $R^{2}$) further strengthened interpretability, showing that fatigue explained up to ∼31% of the variance in key features (e.g., acc_z_mean). These findings demonstrate that IMU stride sequences encode statistically meaningful fatigue signatures that are both large in magnitude and robust across individuals.

### 6.2. Limited Generalization of Global Models Highlights Inter-Individual Variability

In contrast with the clear population-level effects, global LOPO models achieved modest and highly variable accuracy across participants. This outcome is consistent with prior reports on wearable sensor biomechanics and confirms that stride-level running patterns exhibit strong inter-individual specificity. Even with 64 carefully engineered spectral–entropy and time-domain features, global classifiers struggled to generalize to unseen runners. This discrepancy between strong statistical fatigue effects and weak LOPO classification performance underscores a key insight: population-level biomechanical signatures do not translate directly into population-level predictive models. Individualized neuromuscular strategies, anatomical differences, posture, and running styles collectively limit the transferability of global fatigue models.

### 6.3. Personalized Supervised Models Achieve Near-Perfect Performance

When models were trained within participants, supervised personalized Random Forest classifiers achieved exceptionally high performance (mean accuracy 97.7%; mean AUC 0.997). This near-perfect separability confirms that fatigue alters stride biomechanics in consistent and individualized ways that are not preserved under cross-participant pooling. These results show that personalized calibration is essential for applications requiring high reliability, such as injury-risk monitoring [50], training-load management, or athlete-specific coaching systems. They also highlight the limitations of black-box deep learning approaches that do not incorporate individualized structure or interpretable biomechanical features.

### 6.4. NF-Only Personalized Anomaly Detection Supports Minimal Calibration Workflows

The NF-only One-Class SVM models demonstrated that fatigued strides can be reliably detected as deviations from a runner’s NF baseline, even without any fatigued training data. With a mean AUC of 0.967, this method enables real-world deployments where collecting labeled fatigued data is impractical. Conceptually, this aligns with bioinformatics-style anomaly detection, where deviations from a canonical sequence represent functional alterations. Here, fatigued movement patterns behave as “deviations” of an individual’s normal stride structure. This paradigm offers a lightweight, interpretable, and calibration-efficient alternative to supervised learning.

### 6.5. Entropy and Variability Metrics Capture Neuromuscular Degradation

Entropy and stride-to-stride variability analyses provided additional mechanistic insight into fatigue-induced changes. Fatigue generally increased variance, coefficient of variation, and SampEn, particularly for acceleration magnitude features. These findings are consistent with fatigue-induced reductions in rhythmic stability and neuromuscular control. The feature-specific differences—such as stronger entropy increases in acceleration-derived metrics than in gyroscope-derived metrics—suggest that fatigue does not affect all neuromuscular pathways equally. Instead, runners adopt individualized compensatory strategies, as reflected in the heterogeneity observed in the gyroscope-based entropy changes. From a neuromuscular control standpoint, the observed increase in entropy with fatigue likely reflects a shift toward less predictable motor output. As fatigue progresses, altered motor unit recruitment, delayed afferent feedback, and reduced synchronization between agonist and stabilizing muscles may lead to noisier and less regular stride patterns. Such changes are consistent with motor control variability theories, which suggest that fatigue constrains the nervous system’s ability to maintain stable and repeatable movement trajectories. In this context, higher sample entropy indicates a loss of temporal structure and diminished fine-grained control rather than random noise, providing a physiologically meaningful marker of fatigue-induced neuromuscular degradation. These observations further support the interpretation of entropy-based IMU features as surrogate markers of sensorimotor adaptability rather than purely statistical descriptors and align with broader evidence that proprioceptive alterations and sensorimotor adaptation play a central role in fatigue-related movement variability [8].

### 6.6. A Bioinformatics-Inspired Perspective Enhances Sensitivity and Interpretability

The fatigue detection is conceptually analogous to deviation or mutation detection in biological sequences, where departures from an individual baseline carry more discriminative information than absolute population-level patterns. Treating stride windows as structured sequences enabled the application of spectral–entropy, SampEn, and bandpower measures typically used in genomics and physiological-signal analysis. This perspective allowed for the detection of motif-like changes and nonlinear irregularities that are not captured by traditional biomechanical metrics. The integration of multilevel modeling, interpretable spectral–entropy features, and personalized ML represents a methodological advancement over existing IMU fatigue studies, which often rely on narrow feature sets, within-subject evaluation only, or black-box deep networks with limited interpretability.

### 6.7. Practical Implications for Wearable Monitoring and Sports Performance

The results demonstrate that a single lumbar-mounted IMU can provide a rich representation of fatigue-related changes in trunk biomechanics. Global models offer statistically meaningful insight into population-level movement adaptations, while personalized models—especially minimal-calibration NF-only detectors—are best suited for real-time athlete monitoring, coaching feedback, digital twins, and adaptive training systems. Altogether, the proposed framework establishes a scalable and statistically grounded pathway from biomechanical interpretability to deployable, personalized AI systems, offering a scalable blueprint for next-generation wearable fatigue analytics.

## 7. Limitations and Future Work

All experimental design choices and data acquisition procedures reflect those of the original dataset and were not modified or extended in the present analysis. Despite the methodological breadth and strong empirical results, several limitations must be acknowledged to contextualize the findings and guide future research. First, the study relies on a single dataset collected from nineteen recreational runners using a lumbar-mounted IMU. Although mixed-effects modeling confirmed robust fatigue effects across participants, the limited sample size and homogeneous cohort restrict the generalizability of the global models. Future work should validate the proposed framework on larger and more diverse populations, including elite athletes, sport teams [51], different age groups, and clinical populations with altered gait patterns. Second, while the present study focuses on a single sensor location, fatigue manifests through multi-joint adaptations that may be more comprehensively captured using multi-IMU configurations or complementary sensing modalities (e.g., EMG; footstrike pressure insoles). Evaluating how multi-sensor fusion improves entropy-based or sequence-level modeling represents an important next step. Third, the beep-test protocol induces substantial metabolic and neuromuscular fatigue, but it captures only one dimension of athlete fatigue. Additional protocols—such as prolonged submaximal running, interval-based fatigue, or cognitive–motor dual-task fatigue—should be investigated to evaluate whether the entropy- and variability-based signatures generalize across fatigue types. Fourth, global machine learning models demonstrated limited generalization (LOPO accuracy ≈0.55), highlighting pronounced inter-individual variability in running biomechanics. Although personalized classifiers and NF-only anomaly detection provided excellent performance, they require participant-specific data for calibration. Developing hybrid transfer-learning approaches, personalized model initialization, or meta-learning strategies may reduce the dependence on intensive per-subject calibration. Finally, entropy, bandpower, and stride-to-stride variability metrics were extracted using fixed parameter choices (e.g., SampEn parameters; FFT windowing). Adaptive or data-driven parameter optimization could further improve sensitivity to fatigue-induced micro-dynamics, and real-time implementations must consider computational efficiency and hardware constraints. Overall, future work will focus on validating the proposed framework across broader populations, evaluating alternative fatigue paradigms, integrating multimodal wearable data, and developing adaptive or real-time personalized fatigue-monitoring models suitable for embedded or edge-computing systems. Several of the methodological limitations identified in this study are inherited from the publicly available dataset used for analysis. Future extensions of this research will involve the collection of new experimental data using a fully standardized acquisition protocol designed to reduce sources of measurement uncertainty identified in the present dataset. In particular, sensor placement will be explicitly standardized by positioning a single IMU centrally over the lumbar spine, aligned with the midline at approximately the L3–L5 vertebral level using palpable anatomical landmarks (e.g., iliac crest line), and secured with an elastic belt to minimize relative motion. All sensors will be mounted by the same trained experimenter following a consistent protocol, enabling assessment of inter-session and inter-rater reliability. Such a protocol will allow for systematic evaluation of placement variability effects and further strengthen the robustness and reproducibility of entropy- and variability-based fatigue detection in real-world running scenarios.

### Ethical Considerations

This study was conducted in full compliance with institutional, national, and international ethical standards for research involving human participants. The experimental protocol, including participant recruitment, data collection procedures, and IMU instrumentation, was reviewed and approved by the Human Research Ethics Committee at University College Dublin (UCD), ensuring adherence to the principles outlined in the Declaration of Helsinki. All participants were recreational runners, injury-free at the time of recruitment, and engaged voluntarily in the study procedures. The methodological contributions of this study may eventually inform real-time athlete monitoring, rehabilitation support, or occupational fatigue detection. While these applications have potential benefits, careful consideration is required to ensure that such systems are not used for surveillance, coercion, or performance pressure without adequate consent and oversight. Ethical deployment of wearable-based fatigue analytics should always prioritize the autonomy, well-being, and privacy of individuals. Overall, this research complied with all relevant ethical standards and demonstrates the responsible collection, processing, and interpretation of human sensor data within a scientific context.

## 8. Conclusions

This study introduced a unified, bioinformatics-inspired framework for IMU-based fatigue detection that combines mixed-effects statistical modeling, entropy- and spectral-based sequence characterization, stride-to-stride variability metrics, and hybrid machine learning approaches. Using a single lumbar-mounted IMU, we demonstrated that fatigue induces consistent, multidimensional, and statistically robust alterations in trunk acceleration and rotational dynamics across runners. Effect size analyses, including partial $R^{2}$ values ranging from 0.12 to 0.31, confirmed that fatigue explains a substantial proportion of variance in key biomechanical features, while increases in variability and SampEn highlight diminished neuromuscular control under fatigued conditions. Global population-level classifiers evaluated with LOPO cross-validation achieved only moderate accuracy, underscoring the considerable inter-individual variability in running biomechanics and fatigue responses. In contrast, personalized learning approaches—both supervised Random Forest models and NF-only anomaly detectors—provided highly accurate within-athlete fatigue classification, frequently approaching perfect discrimination (AUC ≈ 1.0). These findings confirm that fatigue signatures are strongly individual-specific and that personalized calibration is essential for reliable monitoring in real-world environments. By integrating entropy, spectral features, and variability metrics, the proposed framework captures subtle biomechanical adaptations that are often invisible to traditional time-domain measures alone. From a deployment perspective, the bioinformatics-inspired framework is well suited for real-time wearable applications. The extracted spectral–entropy and variability features are computationally lightweight and can be computed online on embedded processors or mobile devices. Personalized calibration strategies, such as NF-only baseline learning or short supervised warm-up sessions, enable rapid individual adaptation without requiring extensive labeled fatigue data. These properties support practical integration into field-based monitoring systems, digital coaching platforms, and athlete-centric decision support tools operating in uncontrolled training and competition environments. Prospective extensions may integrate multimodal sensor data, adaptive online learning strategies, and digital-twin paradigms to support real-time, personalized fatigue analytics and to expand the generalizability and deployment of this framework across heterogeneous movement tasks and environments. All proposed feature extraction and classification steps are computationally lightweight and suitable for real-time or near-real-time execution on embedded or mobile platforms.

## Figures and Tables

**Figure 11:**
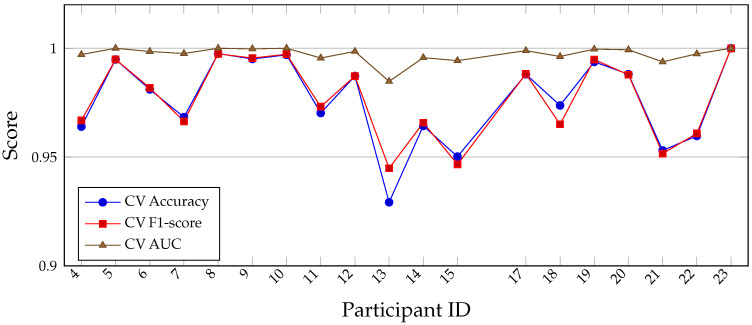
Per-participant performance of supervised personalized RF models using stratified K-fold cross-validation. Accuracy, F1-score, and AUC are consistently high across runners, with several participants achieving near-perfect separation of NF and fatigued strides.

**Table 2:** Mixed-effects regression results for the acceleration magnitude kurtosis (acc_mag_kurt). A Shimmer3 lumbar IMU recorded stride-level signals from 19 participants across NF and F conditions.

Parameter	Coef.	Std. Err.	z	*p* > |z|	2.5%	97.5%
Intercept	0.504	0.391	1.287	0.198	−0.263	1.270
Fatigue	1.430	0.036	39.618	0.000	1.359	1.500
Group Var	2.895	0.678	–	–	–	–

**Table 3:** Mixed-effects regression results for average vertical acceleration (acc_z_mean).

Parameter	Coef.	Std. Err.	z	*p* > |z|	2.5%	97.5%
Intercept	2.199	0.290	7.591	0.000	1.631	2.767
Fatigue	−0.842	0.016	−52.000	0.000	−0.873	−0.810
Group Var	1.592	0.829	–	–	–	–

**Table 4:** Mixed-effects regression results for lateral acceleration peak (acc_y_max).

Parameter	Coef.	Std. Err.	z	*p* > |z|	2.5%	97.5%
Intercept	43.350	2.567	16.887	0.000	38.319	48.382
Fatigue	6.869	0.204	33.637	0.000	6.469	7.269
Group Var	124.787	5.154	–	–	–	–

**Table 5:** Mixed-effects regression results for gyroscope RMS in the lateral axis (gyro_y_rms).

Parameter	Coef.	Std. Err.	z	*p* > |z|	2.5%	97.5%
Intercept	77.275	4.025	19.197	0.000	69.385	85.164
Fatigue	8.358	0.292	28.636	0.000	7.786	8.931
Group Var	306.997	8.889	–	–	–	–

**Table 6:** Comparison of fatigue fixed effects across selected IMU-derived stride features using linear mixed-effects modeling. All models include a participant-level random intercept. Fatigue shows significant influence on all presented features.

Feature	$\beta_{\mathrm{fatigue}}$	Std. Err.	z	*p*-Value	95% CI	Interpretation
acc_mag_kurt	1.4297	0.0361	39.62	<$1\times 10^{-300}$	[1.359, 1.500]	Sharper acceleration peaks (impact kurtosis ↑)
acc_z_mean	−0.8415	0.0162	−52.00	<$1\times 10^{-300}$	[−0.873, −0.810]	Reduced vertical acceleration (propulsion ↓)
acc_y_max	6.8692	0.2042	33.64	$4.82\times 10^{-248}$	[6.469, 7.269]	Increased mediolateral trunk acceleration peaks
gyro_y_rms	8.3584	0.2919	28.64	$2.40\times 10^{-180}$	[7.786, 8.931]	Increased lateral-axis rotational activity

**Table 7:** Effect size measures for the mixed-effects models evaluating the impact of fatigue on selected IMU-derived stride features. Cohen’s *d* quantifies standardized magnitude; $R_{\mathrm{partial}}^{2}$ reflects variance uniquely explained by fatigue.

Feature	Fatigue Coefficient ($\hat{\beta }$)	Cohen’s *d*	Partial $R^{2}$	Interpretation
acc_mag_kurt	1.430	1.03	0.207	Large effect, strong fatigue signal
acc_z_mean	−0.842	1.35	0.310	Very large effect; vertical loading highly fatigue-sensitive
acc_y_max	6.869	0.87	0.158	Moderate–large effect; increased lateral motion
gyro_y_rms	8.358	0.74	0.120	Moderate effect; trunk rotation becomes more variable

**Table 8:** Dataset characteristics, LOPO performance.

Item	Value/Description
Feature matrix shape	(6006 samples, 64 features)
Number of NF strides	2926 strides
Number of fatigued (F) strides	3080 strides
LOPO accuracies (per participant)	[0.4781, 0.5051, 0.7296, 0.5597, 0.3772, 0.5012, 0.4801, 0.4375, 0.5064, 0.3462, 0.4846, 0.5479, 0.5967, 0.8881, 0.4219, 0.4238, 0.6188, 0.6335, 0.8900]
Mean LOPO accuracy	0.5487

**Table 9:** Top 20 most important features from the Random Forest fatigue classifier.

Top 20 Most Important Features	Importance Score
acc_z_mean	0.063114
acc_mag_mean	0.041578
acc_mag_kurt	0.037081
acc_x_mean	0.036126
acc_y_min	0.029727
gyro_y_energy	0.026149
acc_mag_skew	0.025676
gyro_y_rms	0.024405
gyro_y_min	0.023969
gyro_z_std	0.023741
acc_y_kurt	0.021935
gyro_z_energy	0.021153
gyro_z_rms	0.020578
gyro_y_std	0.020149
acc_x_max	0.018366
acc_z_min	0.017617
acc_x_rms	0.017477
gyro_z_skew	0.016155
gyro_mag_energy	0.016079
acc_x_energy	0.015967

**Table 10:** Per-participant performance of the personalized NF-only anomaly detection model (One-Class SVM). Each model is trained on NF strides only and evaluated on both NF and fatigued (F) strides of the same participant.

Participant ID	# Strides	# F	# NF	Accuracy	F1-Score	AUC
4	251	130	121	0.756972	0.738197	0.839987
5	198	98	100	0.949495	0.951456	1.000000
6	318	164	154	0.937107	0.941520	0.991448
7	159	75	84	0.905660	0.909091	1.000000
8	403	201	202	0.967742	0.968675	1.000000
9	407	221	186	0.953317	0.958242	0.996935
10	327	177	150	0.905199	0.911681	0.971337
11	368	207	161	0.907609	0.915423	0.965553
12	235	118	117	0.936170	0.939271	0.995219
13	156	100	56	0.878205	0.905473	0.949286
14	421	222	199	0.786223	0.764398	0.875617
15	261	124	137	0.773946	0.746781	0.869790
17	419	211	208	0.957041	0.958140	0.993894
18	268	104	164	0.951493	0.941176	1.000000
19	320	189	131	0.953125	0.961832	1.000000
20	420	209	211	0.959524	0.960920	1.000000
21	362	176	186	0.906077	0.903409	0.972996
22	322	169	153	0.869565	0.873494	0.950536
23	391	185	206	0.961637	0.961039	1.000000
Mean				0.9061	0.9058	0.9670

**Table 16:** Stride-to-stride variability metrics for selected IMU features [47]. For each participant and fatigue state (F = fatigued; NF = non-fatigued), stride-level feature trajectories were summarized by mean, variance (var), coefficient of variation (CV), and SampEn computed across strides.

Participant	State	Feature	# Strides	Mean	Var	CV	SampEn
4	F	acc_mag_rms	130	20.5342	0.617339	0.038264	2.592341
4	F	gyro_mag_rms	130	130.7319	143.742110	0.091709	1.497421
4	NF	acc_mag_rms	121	20.7230	0.496860	0.034015	1.929103
4	NF	gyro_mag_rms	121	138.8688	129.815416	0.082046	1.583897
5	F	acc_mag_rms	98	20.1692	1.429301	0.059275	2.395080

Note: This is an example for participants 4 and 5, but for coaches the table can be extended to specific/limited number/all participants, both features, and both states.

**Table 17:** Stride-to-stride variability changes between fatigued (F) and NF states for selected participants and features. ΔCV and ΔSampEn are computed as F–NF differences across strides.

Participant	Feature	State	# Strides	CV	SampEn	ΔCV	ΔSampEn
4	acc_mag_rms	F	130	0.0383	2.5923	+0.0043	+0.6632
4	acc_mag_rms	NF	121	0.0340	1.9291		
4	gyro_mag_rms	F	130	0.0917	1.4974	+0.0097	−0.0865
4	gyro_mag_rms	NF	121	0.0820	1.5839		

## Data Availability

The dataset used in this experiment is known as the “Multivariate Time Series data of Fatigued and Non-Fatigued Running from Inertial Measurement Units (0.0) [Data set]” and is freely available for academic research; there are no (legal or other) constraints on using the data for scientific purposes [35]. The data is available upon request. The original contributions presented in the study are included in the article; further inquiries can be directed to the corresponding author.

## Abbreviations

The following abbreviations are used in this manuscript:

IMU	Inertial Measurement Unit
LOPO	Leave-One-Participant-Out
RMS	Root-Mean-Square
ML	Machine Learning
RF	Random Forest
SVM	Support Vector Machines
GB	Gradient Boosting
1D-CNNs	One-dimensional Convolutional Neural Networks
NF	Non-Fatigued
FFT	Fast Fourier Transform
PSD	Power Spectral Density
SaEn	Sample Entropy
AI	Artificial Intelligence
NN	Neural Network
DL	Deep Learning
RL	Reinforcement Learning
